# Antibiotic growth promoters enhance animal production by targeting intestinal bile salt hydrolase and its producers

**DOI:** 10.3389/fmicb.2014.00033

**Published:** 2014-02-11

**Authors:** Jun Lin

**Affiliations:** Department of Animal Science, The University of TennesseeKnoxville, TN, USA

**Keywords:** antibiotic growth promoters, bile salt hydrolase, *Lactobacilli*

## Abstract

The growth-promoting effect of antibiotic growth promoters (AGPs) was correlated with the decreased activity of bile salt hydrolase (BSH), an intestinal bacteria-produced enzyme that exerts negative impact on host fat digestion and utilization. Consistent with this finding, independent chicken studies have demonstrated that AGP usage significantly reduced population of *Lactobacillus* species, the major BSH-producers in the intestine. Recent finding also demonstrated that some AGPs, such as tetracycline and roxarsone, display direct inhibitory effect on BSH activity. Therefore, BSH is a promising microbiome target for developing novel alternatives to AGPs. Specifically, dietary supplementation of BSH inhibitor may promote host lipid metabolism and energy harvest, consequently enhancing feed efficiency and body weight gain in food animals.

## INTRODUCTION

Epidemiological studies have linked usage of antibiotic growth promoters (AGPs) to the emergence of antibiotic resistant bacteria (Wegener, 2003). Antibiotic-resistant bacteria as well as resistance determinants can therefore spread from animals to humans, compromising the effectiveness of antibiotics for treating human infections and posing a serious threat to public health (Wegener, 2003). For this reason, Denmark banned all AGPs in 1998 and European Union member nations banned all AGPs in 2006 (Wegener, 2003; Dibner and Richards, 2005). Therefore, there is a worldwide trend to limit AGP use in food animals (Turnidge, 2004; Dibner and Richards, 2005). Ending the use of AGPs creates challenges for the animal feed and feed additive industries. Several products, such as probiotics, prebiotics, and organic acids have been used to alter intestinal microbiota for improving animal health and production (Dibner and Richards, 2005). However, limited data is available to justify the choice of specific bacterial species or products for such microbiota manipulation. Examination of microbiota in response to AGP treatment would provide insights into the modes of action of AGPs and facilitate the development of more effective microbiota-based strategies for growth promotion. Recent studies on the relationship between AGP usage and gut microbiota strongly suggest that bile salt hydrolase (BSH) is an important target through which microbiome composition and function may impact host fat digestion and energy harvest.

### RESPONSE OF INTESTINAL BSH ACTIVITY TO AGP

Clearly a connection has been made between AGP usage, growth promotion, and intestinal bacterial populations, but the precise mechanism is yet to be delineated as to how these all coincide. Strides have been made in uncovering this mystery, however, and it has been shown that the growth-promoting effect of low-dose antibiotics does coincide with a decrease in BSH activity in the gut (Feighner and Dashkevicz, 1987; Knarreborg et al., 2004; Guban et al., 2006). BSH produced by gut bacteria catalyzes deconjugation of conjugated bile acids (CBAs, also referred to as conjugated bile salts) in the intestine (Begley et al., 2006). CBAs consist of a hydrophobic steroid core that is conjugated with either glycine or taurine. Thus, the CBAs are amphipathic and function as a more efficient “biological detergent” than unconjugated bile acids to emulsify and solubilize lipids for fat digestion (Begley et al., 2006). Consequently, BSH activity has significant impact on host physiology by disturbing CBA-mediated fat metabolism and endocrine functions (Begley et al., 2006; Jones et al., 2008).

Feighner and Dashkevicz (1987) provided early evidence that antibiotic feed additives affect the transformation potential and hydrolysis activity of BSHs from intestinal contents of poultry. By keying in on a more specific aspect of lipid metabolism, Knarreborg et al. (2004) demonstrated an enhanced bioavailability of α-tocopheryl (alpha-tocopherol) acetate in broilers given AGPs, and this was attributed to a reduced concentration of unconjugated bile salts. Furthermore, Guban et al. (2006) correlated dietary supplementation of AGPs to improved weight gain and fat digestibility in broilers, decreased population levels of *Lactobacillus salivarius*, and a reduced pool of deconjugated bile salts. Based on these discoveries, the growth-promoting effect of AGPs likely is partly attributed to the reduced BSH activity, and thus the improvement of host lipid metabolism.

### RESPONSE OF INTESTINAL MICROBIOTA TO AGP

With the aid of culture-independent molecular approaches, the investigations of the effect of AGPs on intestinal microbiota have been initiated in different food animals, including poultry and swine, which greatly improves our understanding of intestinal microbiota changes in response to AGPs (Engberg et al., 2000; Knarreborg et al., 2002; Collier et al., 2003; Dumonceaux et al., 2006; Guban et al., 2006; Wise and Siragusa, 2007; Zhou et al., 2007; Danzeisen et al., 2011; Kim et al., 2012; Lin et al., 2013). As expected, oral administration of low-dose antibiotics affected diversity and relative abundance of gut microbiota in all these studies. However, based on the findings from these molecular ecology studies, it is challenging to definitively link specific bacterial populations to the enhanced growth performance due to AGP usage. Interestingly, independent studies (Engberg et al., 2000; Knarreborg et al., 2002; Dumonceaux et al., 2006; Guban et al., 2006; Zhou et al., 2007; Danzeisen et al., 2011; Lin et al., 2013) showed that AGP usage significantly reduced the population of *Lactobacillus* species, a major bacterial choice for probiotics development; in particular, population of *L. salivarius*, the dominant lactic acid bacterium present in the chicken intestine, was reduced in response to AGP treatment. Since *Lactobacillus *populations are major BSH-producers in small intestine (Begley et al., 2006), this interestingly bacterial shift due to AGP usage is consistent with the reduced BSH activity as described above. Taken together, these studies indicate that the AGP-mediated body weight gain in food animal is inversely related to the activity of BSH enzymes and the abundance of corresponding *Lactobacilli* producers.

### CHARACTERISTICS OF BSH

Bacterial BSH is a member of the choloylglycine hydrolase family of enzymes and is predominantly associated with gastrointestinal bacteria of both humans and animals. Additionally, it is classified as an N-terminal nucleophilic hydrolase and can recognize substrate at both the amino acid conjugate or steroid nucleus (Patel et al., 2010). BSH is particularly abundant in lactic acid fermenting probiotic strains like *Lactobacilli* and *Bifidobacteria* (Begley et al., 2006). BSH enzymes display either narrow or broad substrate specificity; most BSH enzymes from lactic acid bacteria showed higher catalytic abilities to hydrolyze glyco-CBAs than tauro-CBAs (Begley et al., 2006). Despite recent significant progress in the characterization of diverse BSH enzymes, research on BSH is still in its infancy (Patel et al., 2010). The natural functions of BSH enzymes for bacteria themselves are still not clear (Begley et al., 2006). One hypothesis is that BSH activity confers *Lactobacilli* tolerance to bile in the intestine (Begley et al., 2006). However, there are contradictory reports about the correlation between bile tolerance and BSH activity in intestinal bacteria (Begley et al., 2006). For example, production of BSH does not determine bile resistance level in *L. salivarius*, the dominant *Lactobacillus* species present in the chicken intestine (Fang et al., 2009). Regardless the natural function of BSH for its bacterial producer, it has been increasingly recognized that intestinal BSH plays an important role in host lipid metabolism and energy harvest (Begley et al., 2006; Jones et al., 2008). Recent probiotics studies have already shown that oral administration of BSH-producing *Lactobacilli* could affect lipid metabolism, consequently lowering cholesterol level in humans (Jones et al., 2012), rats (Kumar et al., 2011), and pigs (De et al., 1998), which is likely mediated through BSH activity. In the future, more functional, genomic, and microbiological studies are needed to better understand the role of BSH in the symbiosis relationship between gut microbiota and host.

### PRODUCTION OF BSH BY PROBIOTICS: A NEGATIVE TRAIT FROM ANIMAL PRODUCTION PERSPECTIVES?

It is no doubt that dietary probiotics, the normal commensal microorganisms, could exert various beneficial effects on food animals. However, probiotics do impose a variety of potential costs (or detrimental effects) to the animal host as well, which include the production of toxic metabolites, decreased fat digestibility due to production of BSH, and the increase of mucus secretion and gut epithelial cell turn-over (Gaskins et al., 2002; Dibner and Richards, 2004, 2005). Due to such opposite impact of probiotics on the host, it is not surprising that inconsistent results on growth performance of poultry have been observed following probiotic administration (Perumalla et al., 2011). In particular, results available from the literature on probiotic treatments often appear to be contradictory. *Lactobacilli* have been a major bacterial choice for probiotic development in poultry. In general, dietary probiotic supplementation in chicken increases intestinal populations of *Lactobacilli* (Smirnov et al., 2005). Since *Lactobacilli* are dominant BSH-producers in the intestine, dietary *Lactobacilli* treatment may negatively affect lipid metabolism and energy harvest, consequently imposing negative impact on body weight gain. Recently, two research groups (Mountzouris et al., 2010; Sharifi et al., 2012) have reported that probiotic supplementation to diets significantly reduced body weight gain, fat digestibility, and feed conversion in broilers; in these studies, two different commercially available probiotics with a mixture of various organisms (Protexin and PoultryStar) were used. Based on these findings, the authors have proposed that the detrimental effects of the probiotics on chicken growth are likely attributed to the production of intestinal BSH by *Lactobacilli* (Mountzouris et al., 2010; Sharifi et al., 2012). Therefore, overall beneficial effects associated with specific probiotics should be carefully evaluated. Understanding the science of potential negative traits of probiotics can help us develop “negative-traits-mitigation” strategy (e.g., BSH inhibitors) to optimize probiotic products for enhanced growth performance of food animals and profitability of feed additive industry.

### DISCOVERY OF BSH INHIBITORS

Based on the information reviewed above, BSH inhibitors are promising alternatives to AGP for enhanced production of food animals. This hypothesis has been partly supported by our recent study (Wang et al., 2012) in which a BSH enzyme with broad substrate specificity from a chicken *L. salivarius* strain was characterized. Examination of a panel of dietary compounds identified copper and zinc compounds as potent BSH inhibitor (Wang et al., 2012); notably, copper and/or zinc have been used at high concentrations to aid in feed efficiency and growth promotion in poultry (Ewing et al., 1998; Miles et al., 1998; Arias and Koutsos, 2006) and swine (Smith et al., 1997; Hill et al., 2000; Armstrong et al., 2004). However, long-term use of high doses of copper or zinc in animal feed has raised some serious concerns, such as copper/zinc toxicosis and environmental contamination. Therefore, discovery of potent, safe, and cost-effective BSH inhibitors is highly warranted.

In our recent study (Smith et al., 2014), a rapid and convenient high-throughput screening (HTS) system was developed and has been successfully used for identification of BSH inhibitors. This HTS strategy is based on the unique feature of BSH enzyme: hydrolysis of soluble unconjugated bile salts by BSH generates insoluble unconjugated bile salts that could form significant precipitations (Smith et al., 2014). After optimizing various screening conditions, a pilot HTS was performed using a small compound library comprised of 2,240 diverse compounds, leading to the identification of several promising BSH inhibitors with potential as alternatives to AGPs, such as riboflavin and phenethyl caffeate (Smith et al., 2014). In the future, larger scale HTS may reveal more novel BSH inhibitors. In addition, comprehensive animal trials are needed to determine the effect of “champion” BSH inhibitors on growth performance of food animals.

Interestingly, this HTS study also identified a panel of antibiotics as BSH inhibitor, such as various tetracycline antibiotics and roxarsone that have been widely used as AGPs in food animals (Smith et al., 2014). This unexpected finding suggests a new mode of action of low-dose antibiotics for promoting animal growth: some AGPs may exert their growth-promoting effect on food animals by direct inhibition of intestinal BSH enzymes for enhanced lipid metabolism and energy harvest. However, some AGPs, such as bacitracin that has been widely used in poultry industry (Chapman and Johnson, 2002), only displayed low or little inhibitory effect on BSH activity (Smith et al., 2014). The study by Smith et al. (2014) further showed the complexity of the modes of action of AGPs and provided new insights into the interactions between low-dose antibiotics and gut microbiome.

### CONCLUSIONS AND FUTURE DIRECTIONS

Independent studies have shown the link between usage of AGPs and reduced BSH activity as well as reduced population of some *Lactobacillus *species, the major BSH-producers, in the intestine of food animals. In light of these findings, BSH enzyme is a promising microbiome target for developing novel alternatives to AGPs for enhancing the productivity and sustainability of food animals. Recent characterization of a broad-spectrum BSH from *L. salivarius* (Wang et al., 2012) and development of an efficient HTS system for discovery of BSH inhibitors (Smith et al., 2014) have laid a solid foundation for us to develop BSH inhibitors-based feed additives to replace AGPs. In addition, the findings from screening of BSH inhibitors (Smith et al., 2014) suggest a new mode of action of low-dose antibiotics (direct inhibition of intestinal BSH) for promoting animal growth. In the future, large animal trials are needed to determine the effects of BSH inhibitors on growth performance of food animals. Given that research on BSH is still in its infancy, research on BSH ecology of BSH enzymes in the intestine and the role of BSH in host physiology is also highly warranted in the future.

## Conflict of Interest Statement

The author declares that the research was conducted in the absence of any commercial or financial relationships that could be construed as a potential conflict of interest.
